# Qcorp: an annotated classification corpus of Chinese health questions

**DOI:** 10.1186/s12911-018-0593-y

**Published:** 2018-03-22

**Authors:** Haihong Guo, Xu Na, Jiao Li

**Affiliations:** Institute of Medical Information / Medical Library, Chinese Academy of Medical Sciences & Peking Union Medical College, Beijing, China

**Keywords:** Health Question, Annotation, Classification, Question Answering, Chinese

## Abstract

**Background:**

Health question-answering (QA) systems have become a typical application scenario of Artificial Intelligent (AI). An annotated question corpus is prerequisite for training machines to understand health information needs of users. Thus, we aimed to develop an annotated classification corpus of Chinese health questions (Qcorp) and make it openly accessible.

**Methods:**

We developed a two-layered classification schema and corresponding annotation rules on basis of our previous work. Using the schema, we annotated 5000 questions that were randomly selected from 5 Chinese health websites within 6 broad sections. 8 annotators participated in the annotation task, and the inter-annotator agreement was evaluated to ensure the corpus quality. Furthermore, the distribution and relationship of the annotated tags were measured by descriptive statistics and social network map.

**Results:**

The questions were annotated using 7101 tags that covers 29 topic categories in the two-layered schema. In our released corpus, the distribution of questions on the top-layered categories was treatment of 64.22%, diagnosis of 37.14%, epidemiology of 14.96%, healthy lifestyle of 10.38%, and health provider choice of 4.54% respectively. Both the annotated health questions and annotation schema were openly accessible on the Qcorp website. Users can download the annotated Chinese questions in CSV, XML, and HTML format.

**Conclusions:**

We developed a Chinese health question corpus including 5000 manually annotated questions. It is openly accessible and would contribute to the intelligent health QA system development.

**Electronic supplementary material:**

The online version of this article (10.1186/s12911-018-0593-y) contains supplementary material, which is available to authorized users.

## Background

Seeking health-related information is one of the top activities of today’s online users via both personal computers and mobile devices. 59% of the U.S. adults have looked online for health information in 2012[1]. China has 194.76 million Internet health users in 2016 [2], increased 28.0% compared with that in 2015 [3], and will be further stimulated by the development of the Internet and communication technologies, as well as China’s “Internet Plus” and “Health Big Data” policies[4, 5]. Despite the widespread need, the search engines often failed in returning relevant and trustworthy health information [6, 7]. Automatic question answering (QA) systems that can comprehend the questions asked by users in natural language and respond with concise and correct answers using natural language processing techniques shall be a good way to solve this problem [8]. Therefore, several efforts have worked on exploring automatic QA systems in health and medical area in recently years [9–14]. However, it is challenging [15–17], one of the main challenges is the lack of large scale corpus of annotated questions for the machines to learn to extract and understand the main information needs from the questions, known as question processing, which will obviously affect the performance of a QA system [18].

Due to the significant roles of annotated questions for QA system research and development, several studies have focused on this task and collected some useful corpus. For example, the National Library of Medicine of the United States has collected a total of 4,654 annotated clinical questions [19] via endeavored studies [20–25]. This corpus has been applied for training machines to automatically classify question types [26], distinguishing answerable and unanswerable questions [27], recognizing question entailment [28], extracting keywords of the questions [26], as well as separating consumer questions from clinical questions [29]. Other groups have annotated several small scale corpora of health care-related questions in English, so as to automatically identify questions that can be answered by specific EMR notes [30], analyze the user’s demographic, cognitive, affective, situational, social environmental information that are implied in the questions [31], classify the types of consumer health questions [32–35], and extract structured information from EHR-related ICU questions [36] and so on. These previous studies on English question corpora have provided useful references for Chinese health question corpus development.

Several studies on Chinese health questions corpus development have been conducted. Yin JW [37] annotated 1,600 Chinese questions related to maternal and infant health care with 8 topics so as to conduct automatic question classification. Zhang N [38] annotated 4,465 Chinese questions related to skin diseases with a self-developed two-layered classification schema so as to automatically classify question topics and help computing their semantic similarity. Tang GY [39] manually classified 1,688 questions related to hyperlipidemia into 241 categories in order to computing their semantic similarity. Compared with the above studies, our corpus featured as: (1) its annotation schema covers a large range of health topics; (2) the annotated questions covered broad diversity of diseases; moreover, (3) the corpus is openly accessible and easily reusable. Our work would help the intelligent system development related to Chinese health QA.

In this paper, we presented the Qcorp database which collects annotated health care-related questions in Chinese on the basis of our previous works [40, 41]. In current release, Qcorp contains 5000 consumer health questions in Chinese that are annotated with 7101 tags by 8 annotators with a two-layered classification schema consisting of 29 topic categories. An empirical study conducted by us [41] showed that the corpus was useful in training machines to automatically assign the topics of consumer health questions. We have made the current Qcorp publicly available and would enrich it in future work/collaboration, thus, the corpus could be more useful and applicable in various scenarios.

## Methods

### Data collection

The Qcorp database contains 5000 Chinese consumer health questions in total, and they are divided into two parts: data set 1 is the 2000 hypertension related questions collected by our previous work [40, 41], and data set 2 includes 3000 questions we randomly selected from 5 Chinese health websites under 6 broad sections: internal medicine, surgery, obstetrics & gynecology, pediatrics, infectious diseases, and traditional Chinese medicine (Table 1). These websites were selected because of their disease coverage and data accessibility, and a brief introduction of each of the five websites according to their *about us* section can be found in Additional file 1. The YYNET website lacks traditional Chinese medicine section, and the MYB website lacks either chirurgery or traditional Chinese medicine sections. Thus, no such section questions were collected from these two websites.

**Table 1:** Sources of the 3000 questions in data set 2

Website	Internal medicine	Surgery	Obstetrics & Gynecology	Pediatrics	Infectious Diseases	Traditional Chinese Medicine	Total
HAODF	100	125	100	100	100	170	695
XYWY	100	125	100	100	100	165	690
120ASK	100	125	100	100	100	165	690
YYNET	100	125	100	100	100	N/A	525
MYB	100	N/A	100	100	100	N/A	400
Total	500	500	500	500	500	500	3000

Here, the “question” is defined as a request on a certain subject posted by a consumer via the Internet to elicit answers from the physicians or the patient support group, which was identified based on meaning, not form. We manually discarded the uncomplicated data, repeated data and irrelevant data, such as advertisements, health education contents, patients’ experiences, and other non-health contents. When one question was excluded we randomly selected another question from the same website within the same section so as to keep the sample balance.

### Annotation tasks

Since the various consumer health questions could be represented by limited topics and keywords, the question classification plays an important role in an automatic QA system in identifying the information needs of consumers and further improving the accuracy of returned answers. Here, we performed manual annotation of the general topics of 5000 Chinese questions related to health care posted by consumers via the internet, for the purpose of building a high quality annotated corpus for question classification, and further promoting the research and development of intelligent Chinese health QA systems.

### Annotation guidelines

**Fig. 1:**
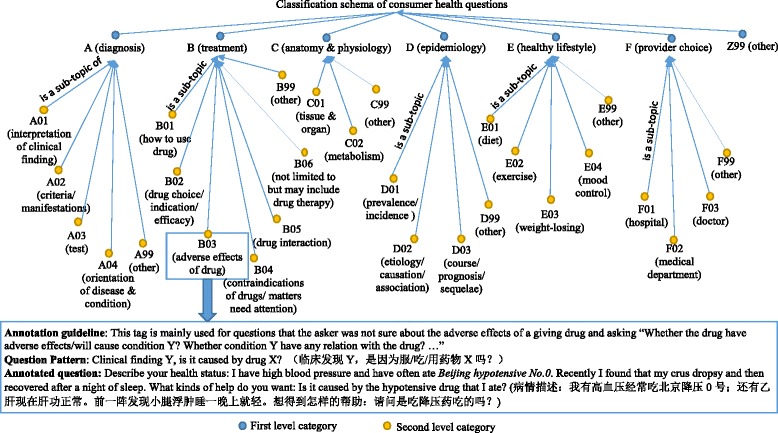
Classification schema of consumer health questions and an example of the corresponding annotation rules

On basis of our previous work [40, 41], we used a two-layered *Classification Schema of Consumer Health Questions* in this study. As shown in Fig. 1, the schema consists of 7 broad categories on the first layer and 28 subcategories on the second layer, where, each category was coded by a unique identifier. The subcategories of other under each broad category were coded as main code plus 99, so as to keep the expandability of the classification schema. To guide the annotation and assure the inter-annotator agreement, we also built a list of annotation rules and question patterns for each category of the classification schema. More details of annotation guidelines can be found at our Qcorp website [42].

### Annotators

We recruited eight annotators, one half of them have medical education background and the other half are specialized in medical informatics. For the 2000 hypertension related questions, their annotations were completed by five annotators in our previous work [41]. We translated their tags into two-layered tags according to the annotation guidelines in this study. For the rest 3000 questions (i.e., internal medicine, surgery, obstetrics & gynecology, pediatrics, infectious diseases, and traditional Chinese medicine), the annotation processes were performed in 3 rounds: In round 1, a training set of 300 randomly selected Chinese questions related to health care were annotated by four annotators independently so as to conclude and modify the annotation guidelines, ambiguous questions were settled by specifying the annotation rules and the question patterns. Then the four annotators were divided into two groups. In round 2, a testing set of 600 questions randomly selected from the sample were assigned to the two groups, 300 questions for each, and each annotator annotated independently so as to measure the inter-annotator agreement. In round 3, a development set of the remaining 2100 questions were each annotated independently by two of the four annotators. The disparities were discussed to achieve an agreement.

### Inter-annotator agreement analysis

The inter-annotator agreement was evaluated by the percentage agreement statistic (Equation 1), which was one of the commonly used metrics to evaluate interrater reliability, and was directly interpretable. As a health question tend to have multiple topic tags, a match was recorded if two annotators agreed with either main or minor tag assignment based on the assumption that it was acceptable to answer any one of them. Therefore, the kappa statistic that focuses on the inter-annotator agreement on each specific category [43] might not meet the multiple tag assignment measurement. This study used as many as 29 topic categories in annotation, thus, the percentage agreement statistic was more suitable.

1 $$ I=\frac{M}{A} $$

Where *M* is the number of tag matched questions, and *A* is the number of all the annotated questions.

### Database framework and web interface

Data stored in the Qcorp database were managed by using MySQL. Social network map and descriptive statistics were used to calculate and visualize the distribution and relationship of the annotated tags. The web server of Qcorp was developed based on Java. The Qcorp database is freely available at http://www.phoc.org.cn/healthqa/qcorp/.

## Results

### Corpus overview

**Fig. 2:**
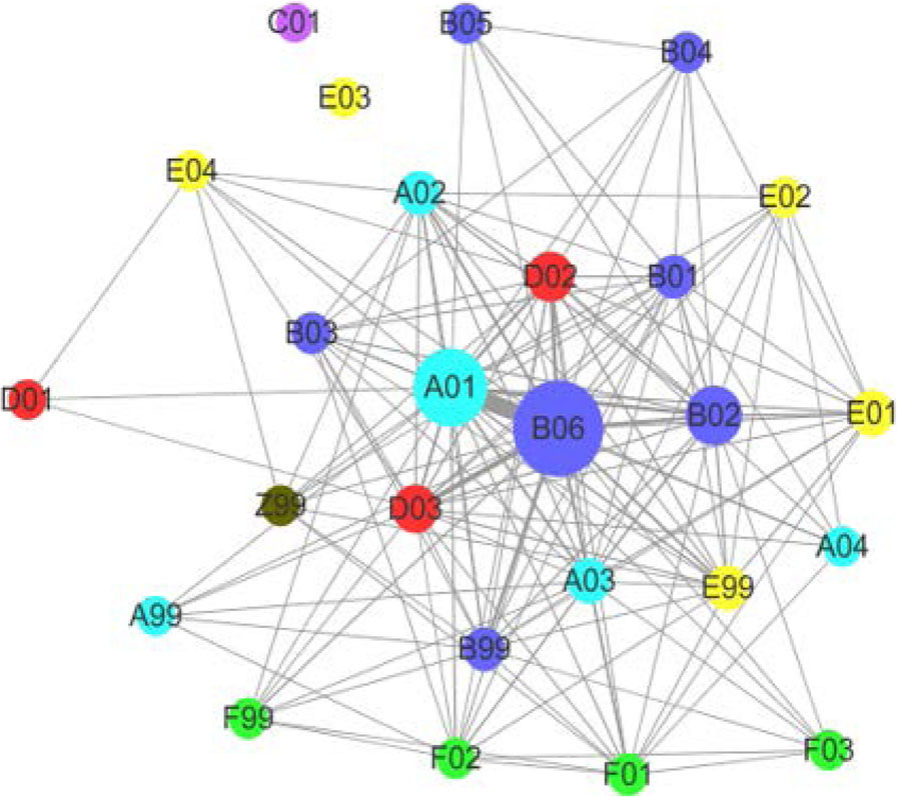
The network of co-annotated tags on consumer health questions. The bigger the node, the more the corresponding tag is annotated; and the thicker the string, the more the two tags are co-annotated. The meaning of the tags were presented in Fig. 1

The 5000 Chinese consumer health questions were annotated with 5000 major tags and 2101 minor tags by the two-layered classification schema which consists of 29 topic categories (Fig. 1). One third of the questions (1717, 34.34%) were annotated with multiple tags, demonstrating that consumers tend to ask more than one question at a time, and the sub-questions sometimes belong to multiple topic categories [44], indicating that the task to classify the topics of consumer health questions was a multi-label problem [45]. As shown in the network of the co-annotated tags (Fig. 2), the tag B06 (represents treatments that not limited to but may include drug therapy) was annotated the most (2228, 44.56%), and it was often associated with other tags, mainly A01 (represents interpretation of clinical finding), D03 (represents course, prognosis, and sequelae of disease), B02 (represents drug choice, indications and efficacy of drug) and B99 (represents other issues about treatment) and so on. The tag A01 covered the second amount of the sample questions (1511, 30.22%), besides B06, it was often associated with A02 (represents criteria and manifestation of disease), D02 (represents etiology and causation of disease, and the association of risk facts and disease), D03, and A03 (represents test). The tag B02 (represents drug choice, indications and efficacy of drug) annotated the third amount of the sample questions (784, 15.68%), and it was interesting to see that it sometimes associated with E01 (represents diet), which indicated that some people were seeking for diet to help them recovering from ill condition while looking for drug therapy.

### Annotated tag distribution on the first layer

**Fig. 3:**
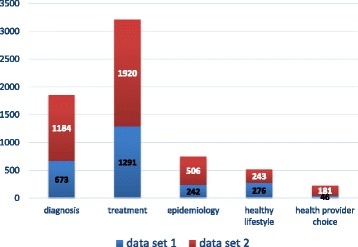
Annotated tag distribution on the five broad categories of consumer health question classification schema. Data set 1 is the 2000 hypertension related questions collected by our previous work, and data set 2 includes 3000 questions we randomly selected from 5 Chinese health websites under 6 broad sections: internal medicine, surgery, obstetrics & gynecology, pediatrics, infectious diseases, and traditional Chinese medicine

For the seven categories on the first layer of the classification schema (Fig. 3), a total of 3211 (64.22%) questions were assigned to treatment, with 64.44% of the data set 1 (1291/2000) and 64.00% of the data set 2 (1920/3000); 1857 (37.14%) questions were assigned to diagnosis, with 33.65% of the data set 1 (673/2000) and 39.47% of the data set 2 (1184/3000); 748 (14.96%) questions were assigned to epidemiology, with 12.10% of the data set 1 (242/2000) and 16.87% of the data set 2 (506/3000); 519 (10.38%) questions were assigned to healthy lifestyle, with 13.80% of the data set 1 (276/2000) and 8.10% of the data set 2 (243/3000); 227 (4.54%) questions were assigned to health provider choice, with 2.30% of the data set 1 (46/2000) and 6.03% of data set 2 (181/3000). There was only one question assigned to anatomy & physiology, but we think this category contains many important knowledge about the basic medicine such as tissues and organs, metabolism and so on, so it was kept in the classification schema. 6 (0.12%) questions and 56 sub-questions could not be assigned to any of the above six broad categories.

### Annotated tag distribution on the second layer

**Fig. 4:**
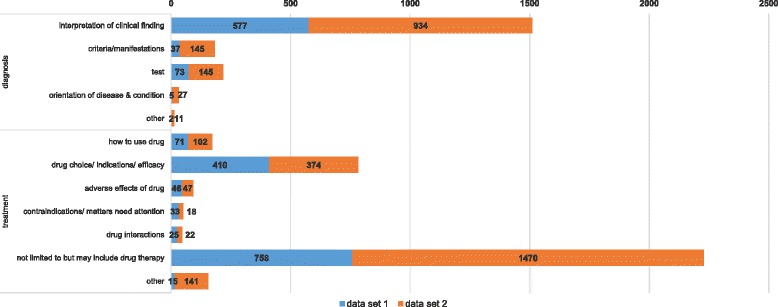
Annotated tag distribution under the categories of diagnosis and treatment. Data set 1 is the 2000 hypertension related questions collected by our previous work, and data set 2 includes 3000 questions we randomly selected from 5 Chi-nese health websites under 6 broad sections: internal medicine, surgery, obstetrics & gynecology, pediatrics, infectious diseases, and traditional Chinese medicine

For questions under the category of diagnosis (Fig. 4), more than 80% of them (1511/1857) were about interpretation of clinical finding, nearly 12% (218/1857) were about test, and almost 10% (182/1857) were about criteria and manifestation of disease. For questions under the category of treatment (Fig. 2.c), almost 70% of them (2228/3211) were seeking treatments that not limited to but may include drug therapy, and nearly one quarter (784/3211) were specified to drug therapy, approximately 6% (173/3211) were about how to use a drug, and lest than 5% (144/3211) were concerned on side effects and contraindications of drugs. Questions under the category of epidemiology were mainly about the course, prognosis, and sequelae of disease (417/748, 55.75%) and etiology and causation of disease, and the association of risk facts and disease (347/748, 46.39%). Half of the Health lifestyle related questions (255/519) were specified to diet, and more than 40% (226/519) were general. For the questions under health provider choice, nearly half (101/227) were asking for a recommendation of hospitals, more than 20% (52/227) were about recommendation of medical departments, about 10% (29/227) were seeking for good doctors, and one quarter (57/227) were asking about the doctor visiting process, doctor appointment and so on.

### Inter-annotator agreement

The inter-annotator agreement for the four annotators on the training set (300 questions) of data set 2 was 0.67 in round 1. By discussing on the disparities and further specifying the annotation rules and the question patterns for each category, the inter-annotator agreement for the two groups on the testing set (300 questions for each group) in round 2 increased to 0.88 and 0.92. After further discussion to achieve an agreement on the disparities, the average inter-annotator agreement for the four annotators on the developing set (2100 questions in total, each was annotated by two annotators independently) in round 3 increased to 0.96. And the average inter-annotator agreement for the five annotators on the data set 1 (each question was at least annotated by two annotators) on the second layer of the classification schema was 0.95.

### Corpus access and usage

#### User interface

**Fig. 5:**
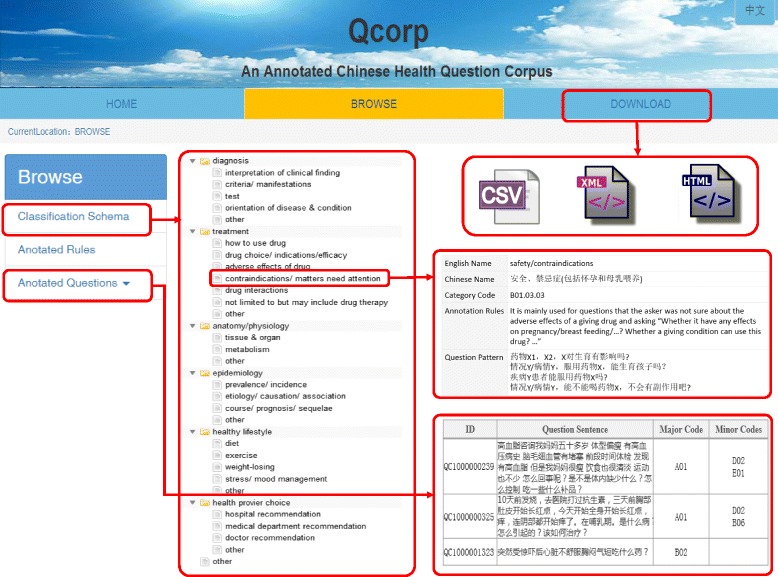
User interface of Qcorp

**Fig. 6:**
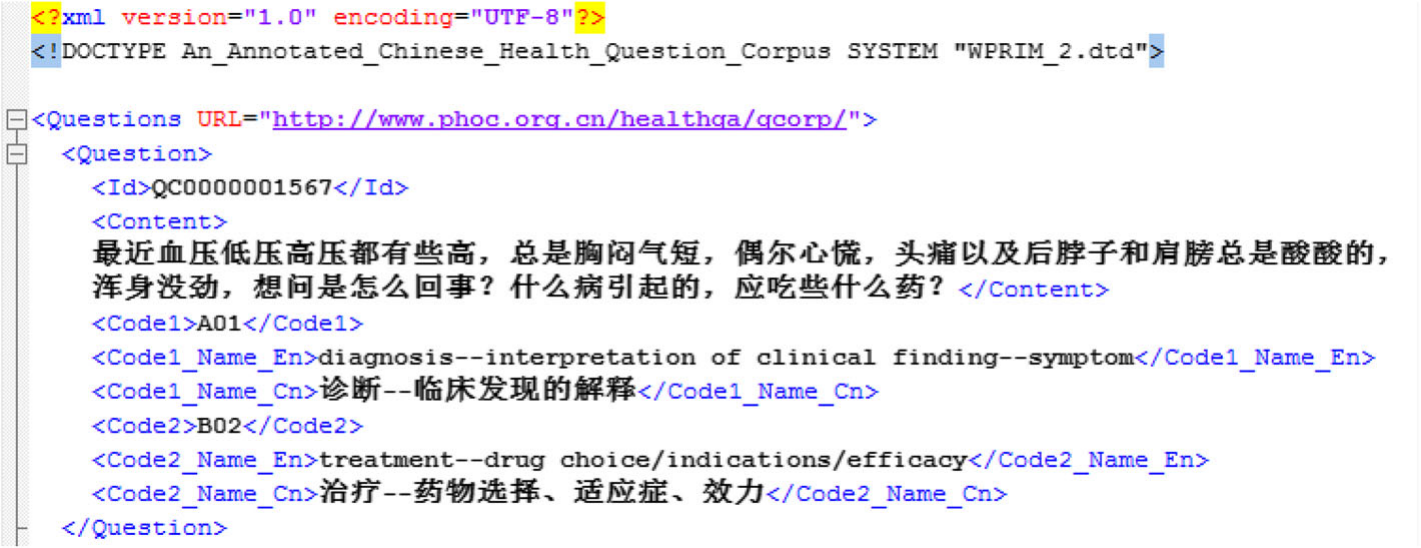
An example of annotated questions in XML format

We provide a user-friendly interface that enable users to access the classification schema, the corresponding annotation rules, as well as the annotated Chinese consumer health questions (Fig. 5). In the “Browse” page, users can browse the classification schema of consumer health questions, and look for the details of each utmost small category with a click, the details of a category include its name in both Chinese and English, category code, annotation rules, question patterns and examples. Users can also browse all the annotation rules and browse the annotated questions by the seven broad categories in the first layer. In the “Download” page, users can download all the annotated Chinese consumer health questions in CSV, XML (Fig. 6 shows an example), and HTML format, so as to use them conveniently according to their usage purposes.

#### Case application of Qcorp corpus

Using the annotated 2000 questions in data set 1 as corpus, we applied a machine-learning method to automatically classify these questions into one of the five topics on the first layer of the classification schema. The Chinese questions were represented as a set of lexical, grammatical, and semantic features, and the features were weighted and selected according to [46]. Among them, Lexical features include bag-of-words and part-of-speech, grammatical features include interrogative words and corresponding chunks, semantic features include the Chinese Medical Subject Headings concepts and semantic types and so on. The result shows that the question classification achieved the F1-score of 99.13%, 98.55%, 96.35%, 76.02%, and 71.77% for the topics of *Healthy Lifestyle*, *Diagnosis*, *Health Provider Choice*, *Treatment*, and *Epidemiology*, respectively (More details can be found in [41]). This demonstrated that these annotated Chinese questions were applicable for training machines to automatically classify the topics of questions posted by health consumers, facilitating answer generation.

## Discussion

### Principle findings

Internet is increasingly becoming one of the main resources for consumers to acquire health information. Automatic QA systems that can correctly answer users’ questions in natural language shall be a promising way to fulfill this need. A shared corpus of annotated consumer health questions in Chinese is prerequisite for training machines to understand the information needs of Chinese consumers by a health QA system. Thus, we developed the Qcorp database which collects annotated health care-related questions in Chinese. Qcorp currently contains 5000 consumer health questions in Chinese that annotated with 7101 tags by 8 annotators with a two-layered classification schema consisting of 29 topic categories. The corpus was proved to be applicable for training machines to automatically assign the topics of Chinese consumer health questions in an empirical study.

### Comparison with other related works

Comparing to other related works on the annotation and corpus building of health and medical questions (Table 2), there are three main specialties in this study. Firstly, the scale of the annotated corpora in our Qcorp database was the biggest. Currently the Qcorp contains 5,000 annotated Chinese health questions, surpass the 4,654 annotated English clinical questions maintained by NLM [19], and the 4,465 annotated Chinese health questions built by Zhang N [38], let alone other small scale corpora. Secondly, the sample questions used in the Qcorp database were randomly selected from multiple sources. Unlike those corpora mainly come from 1 health website [31, 32, 37, 38], our corpus were randomly selected from 5 Chinese health websites so as to improve the representativeness of the corpus. Thirdly, the corpus here covered the relatively more diversity of the diseases. Other similar corpus, especially those Chinese ones, are mainly focused on only one specific kind of diseases, such as genetic and rare diseases [32], cancer [34], maternal and infant diseases [37], and skin diseases [38] and so on. While our corpus were selected from 6 broad sections, including internal medicine, surgery, obstetrics & gynecology, pediatrics, infectious diseases, and traditional Chinese medicine, so as to make it cover as many diseases as possible. To conclude, the Qcorp database is currently the biggest annotated classification corpus of Chinese health questions that from multiple sources and covered relatively more diversity of diseases. Other specialties include that the classification schema modified and applied in this study was quite reliable and with proper layers and number of categories.

**Table 2:** A comparison of works on the corpus building of health and medical questions

Corpus or Author name	Language	Asker	Corpus scale	Question sources	Disease covering	Annotated categories	Layers
NLM collected clinical questions [19]	En	P	4,654	Clinical settings (5 studies [20–25])	Not limited	64	4
Patrick J [30]	En	P	595	Clinical settings	Not limited	11	4
Zhang Y [31]	En	C	600	1 website	23 subcategories	>50	5
Roberts K [32]	En	C	1,467	1 website	Genetic and rare diseases	13	1
Maroy S [34]	En	C	1,279	6 websites	Cancer	10	2
Yin JW [37]	Cn	C	1,600	1 health APP	Maternal and infant health	8	1
Zhang N [38]	Cn	C	4,465	1 website, books, self-composed	Skin disease	52	2
Tang GY [39]	Cn	C	1,688	4 websites	Hyperlipidemia	241	1
Our Qcorp	Cn	C	5,000	5 websites	6 broad sections	29	2

P refers to physician, and C refers to consumer

### Limitations and future studies

The Chinese health question corpus introduced here was only annotated with general topics, and yet was far from precisely representing the health information needs of askers that contained in the questions. There are much work to do to reveal more detailed information of the Chinese consumer health questions in a structured manner. Our next step is to annotate the named entities and their relationships expressed in the Chinese consumer health questions. We hope that this database mainly developed for Chinese consumer health questions could serve as an important resource for the research and development of intelligent Chinese health QA systems.

## Conclusions

We developed a corpus with 5000 Chinese consumer health questions manually annotated using a two-layered classification schema. The corpus, named as Qcorp, was openly accessible with the annotated questions in formats of CSV, XML and HTML, which can be easily used to train machines to understand consumers’ health questions in Chinese. To our knowledge, the Qcorp database is currently the annotated classification corpus of Chinese health questions that covered relatively more diversity of diseases and come from multiple sources. Our study would help Chinese health QA system development.

## Additional file

Additional file 1: A brief introduction of the data source websites. This additional file is in PDF format. It contains a table that gives a brief introduction of the data source websites, including their abbreviation, Chinese name, English names, and description. (PDF 58 kb)

## Abbreviations

AI: Artificial Intelligent
CSV: Comma-Separated Values
HTML: Hyper Text Markup Language
MYB: Manyoubang.com
NLM: National Library of Medicine
QA: Question-answering
XML: Extensible Markup Language
YYNET: YYNET.CN
