# Supplementary material for: Qcorp: an annotated classification corpus of Chinese health questions
Source: BMC Med Inform Decis Mak. 2018 Mar 22;18(Suppl 1):16. doi: 10.1186/s12911-018-0593-y (PMC5872377; doi:10.1186/s12911-018-0593-y)
Supplement: Supplementary file 1 — A brief introduction of the data source websites. This additional file is in PDF format. It contains a table that gives a brief introduction of the data source websites, including their abbreviation, Chinese name, English names, and description. (PDF 58 kb) [file 12911_2018_593_MOESM1_ESM.pdf]

Table 1. A brief introduction about the data source websites

Abbreviation	Chinese Name	English Name	Description
HAODF	好大夫在线	www.haodf.com	HAODF was established in 2006, and have become a leading role in many fields of internet health care, such as online picture and text consultation, telephone counseling, remote outpatient service, appointment referral, and family doctor, and so on. It provides Internet health care information services under the examination and approval of Beijing Municipal Commission of Health and Family Planning.
XYWY	寻医问药	XYWY.COM	XYWY was established in 2004, and have built a complete industry chain and ecosystem of Internet health care by connecting patients, doctors, hospitals, and pharmaceutical companies. Its amount of users and rate of flow kept in the forefront of the Internet health care industry. By the end of 2016, it has more than 120 million registered users, 22 million unique visitors per day, and 320 million unique visitors each month.
120ASK	快速问医生<br>有问必答	www.120ask.com	120ASK is health website hold by Zhuhai Healthcare Cloud Technology Co., Ltd. It aimed at helping health consumers to find the right doctor at any time at anywhere, and providing them with comprehend, timely, favorable and accurate health information.
YYNET	医元网	YYNET.CN	YYNET is the health information service channel of National Public Health Pavilion of China. It brings together many medical experts from multiple disciplines, so as to provide the general public with guidance on health and medicine in a variety of forms and on different levels.
MYB	慢友帮	manyoubang.com	MYB is the biggest chronic disease mutual health social platform in China, aimed at providing a social platform for the patients to share their experiences on drug use and disease treatment, so as to encourage each other, and help each other to improve their quality of life. It won the championship on the new media venture contest in Qingdao in 2014.
